# Working Memory and Cross-Frequency Coupling of Neuronal Oscillations

**DOI:** 10.3389/fpsyg.2021.756661

**Published:** 2021-10-21

**Authors:** Mohammed Abubaker, Wiam Al Qasem, Eugen Kvašňák

**Affiliations:** Department of Medical Biophysics and Medical Informatics, Third Faculty of Medicine, Charles University in Prague, Prague, Czechia

**Keywords:** cross-frequency coupling, neuronal oscillations, phase-amplitude coupling, theta-gamma coupling, working memory

## Abstract

Working memory (WM) is the active retention and processing of information over a few seconds and is considered an essential component of cognitive function. The reduced WM capacity is a common feature in many diseases, such as schizophrenia, attention deficit hyperactivity disorder (ADHD), mild cognitive impairment (MCI), and Alzheimer's disease (AD). The theta-gamma neural code is an essential component of memory representations in the multi-item WM. A large body of studies have examined the association between cross-frequency coupling (CFC) across the cerebral cortices and WM performance; electrophysiological data together with the behavioral results showed the associations between CFC and WM performance. The oscillatory entrainment (sensory, non-invasive electrical/magnetic, and invasive electrical) remains the key method to investigate the causal relationship between CFC and WM. The frequency-tuned non-invasive brain stimulation is a promising way to improve WM performance in healthy and non-healthy patients with cognitive impairment. The WM performance is sensitive to the phase and rhythm of externally applied stimulations. CFC-transcranial-alternating current stimulation (CFC-tACS) is a recent approach in neuroscience that could alter cognitive outcomes. The studies that investigated (1) the association between CFC and WM and (2) the brain stimulation protocols that enhanced WM through modulating CFC by the means of the non-invasive brain stimulation techniques have been included in this review. In principle, this review can guide the researchers to identify the most prominent form of CFC associated with WM processing (e.g., theta/gamma phase-amplitude coupling), and to define the previously published studies that manipulate endogenous CFC externally to improve WM. This in turn will pave the path for future studies aimed at investigating the CFC-tACS effect on WM. The CFC-tACS protocols need to be thoroughly studied before they can be considered as therapeutic tools in patients with WM deficits.

## Introduction to Brain Oscillations and Working Memory

The brain (neuronal) oscillations arise from the simultaneous interactions between the neuronal networks and are divided into five frequency bands: delta (0.5–3.5 Hz), theta (3.5–7 Hz), alpha (8–13 Hz), beta (18–25 Hz), and gamma (30–70 Hz) (Düzel et al., 2010; Başar, 2013; Merker, 2013; Luo and Guan, 2018). The brain oscillations can be detected by scalp electroencephalography (EEG) or directly in the cortex (Electrocorticography, ECoG, or intracranial EEG). Similarly, the neuronal oscillations originating from the sulci can be detected with scalp magnetoencephalography (MEG) (Marzetti et al., 2019; Andersen et al., 2020). The EEG frequency bands reflect the rhythmic and synchronized postsynaptic potentials that arise from the pyramidal neuronal assemblies (Jensen et al., 2014). Moreover, brain oscillations are predictive of information processing and are involved in selective communication and information transmission (Fries et al., 2001; Fries, 2015). The power of the oscillation bands and the coupling between the brain regions constantly change in response to task demands (Klimesch, 2018). For instance, gamma-band synchronization is triggered by stimuli and is essential for cortical computation (Fries, 2009). All the studies suggest an important role of neuronal oscillations in brain functions (Engel et al., 2001; Niebur, 2002; Buzsáki and Draguhn, 2004; Mann and Paulsen, 2005).

The neuronal oscillations play different roles in cognition/psychology: delta bands are associated with deep sleep and long-range coordination between the neuronal networks (Hiltunen et al., 2014; Leszczyński et al., 2015); theta bands are represented in shallow sleep, meditative states, coordination of memory encoding and maintenance (hippocampal theta), and long-range coordination of cognition (cortical theta) (Sederberg et al., 2003; Axmacher et al., 2010; Sauseng et al., 2010; Cohen, 2014); alpha bands are commonly associated with rest, relaxation, memory, and motor inhibition (Sauseng et al., 2009; Roux and Uhlhaas, 2014); beta bands are linked to awareness and attention (Egner and Gruzelier, 2004; Buschman and Miller, 2007). In contrast to delta, theta, and beta oscillations, the high-frequency gamma oscillations arise from the negative feedback between the GABAergic interneurons and pyramidal neurons; gamma oscillatory activities perform different computations and represent different information patterns (Fries et al., 2007; Jensen and Colgin, 2007).

### Cross-Frequency Coupling

Cross-frequency coupling (CFC) is the interaction between the brain oscillations on different frequency bands (Jirsa and Müller, 2013; Sotero, 2016; Siebenhühner et al., 2020). From a theoretical perspective, there are four ways in which CFC can occur: phase-to-amplitude, power-to-power, phase-to-phase, and phase-to-frequency interactions (Jensen and Colgin, 2007; Helfrich et al., 2016). In power-to-power coupling: the changes in the power of the faster oscillations are correlated with the power changes in the lower frequency bands; in phase to phase coupling: phase-locking occurs between oscillations at different frequencies and their phase relationship remains constant; in phase to power coupling: the power of the fast oscillations is modulated by the phase of the slow oscillations (Schack et al., 2002; Bruns and Eckhorn, 2004; Lakatos et al., 2005; Mormann et al., 2005; Canolty et al., 2006). The phase-amplitude coupling (PAC) is a widely observed model of CFC in which the high-frequency amplitudes are modulated by the low-frequency phases (Canolty and Knight, 2010; Siems and Siegel, 2020). Abnormal CFCs have been reported by several studies conducted in patients with Parkinson's disease, Alzheimer's disease (AD), schizophrenia, mental disorders, and anxiety (Allen et al., 2011; De Hemptinne et al., 2013; Alegre, 2016; Lynn and Sponheim, 2016; Wang et al., 2017). The neural modulations/entrainments are classically divided into three approaches: sensory, non-invasive electrical/magnetic, and invasive electrical entrainment (Thut and Miniussi, 2009; Calderone et al., 2014; Herrmann et al., 2016; Hanslmayr et al., 2019).

### Working Memory

Working memory (WM) is the active retention and manipulation of information over a few seconds and is considered an essential component of cognitive function (Aben et al., 2012; Cowan, 2014, 2017; Persuh et al., 2018). Although the storage capacity of WM is inherently limited (Fougnie et al., 2015), several studies have found that the WM capacity can be altered by training (Botvinick and Watanabe, 2007; MacOveanu et al., 2007; Edin et al., 2009). Neural activity in the prefrontal cortex and the strength of connectivity between the prefrontal and parietal cortices have been shown to be improved by training, as suggested by the studies in the humans and non-human primates (Klingberg et al., 2002; Jaeggi et al., 2008; Siegel et al., 2012; Constantinidis and Klingberg, 2016). Training has a primary benefit on tasks that are very similar to the training tasks and does not improve overall WM capacity (Hulme and Melby-Lervåg, 2012). From a theoretical perspective, the two terms have been used extensively to describe the temporal storage of information: Short-term memory (STM) and WM. STM is an essential component for holding motion, sensory, and cognitive information for a short interval of time. STM describes the process of passively maintaining the information over a short period of time, while the WM concept depicts the processes of maintaining and manipulating the information for a short period of time. Thus, information manipulation is the main difference between the two concepts (Aben et al., 2012; Cowan, 2017). Despite the differences between STM and WM, the two terms are still used interchangeably in the literature. It has been suggested that the two concepts represent the same cognitive process (Baddeley, 1992; Gathercole and Alloway, 2006; Unsworth and Engle, 2007; Klingberg, 2010; Nadel and Hardt, 2011). The tasks involving only item maintenance have often been used to test STM, such as word span, digit span, and delayed match-to-sample tasks, while the tasks involving item maintenance and manipulation have classically been used to test WM, such as n-back, computation span, mental control, and letter-number sequencing tasks (Engle et al., 1999; Kane et al., 2004; Ackerman et al., 2005; Conway et al., 2005; Colom et al., 2006). In addition, the mental arithmetic tasks have been considered as the primary tasks in WM assessment, since the solution of problems in these tasks activates the WM components (DeStefano and LeFevre, 2010). All the neuronal oscillations are important for the cognitive and memory processes, particularly theta (Klimesch, 1999; Gathercole et al., 2003; Kane et al., 2007; Hsieh and Ranganath, 2014) and gamma bands (Roberts et al., 2013; Roux and Uhlhaas, 2014). The causal relationship between the brain oscillations and memory processes can be tested by modulating the endogenous brain oscillations and assessing the behavioral effects of such modulation.

Several models have been proposed to illustrate the underlying mechanisms behind WM (Kamiński et al., 2011; Van Vugt et al., 2014; Vosskuhl et al., 2015; Wolinski et al., 2018; Sauseng et al., 2019). Two models were adopted; one model states that each memory item is translated into a fast and transient wave that can be detected electro-physiologically (gamma wave). Several individual gamma waves fit into a single theta cycle and the limited WM capacity can be explained by the finite number of gamma waves that can fit into a single theta cycle (Lisman and Idiart, 1995; Jensen and Lisman, 1996). Moreover, the WM capacity of seven items has been reported in the studies that used immediate verbal recall tasks (Gignac, 2015), while the studies that used rehearsal of verbal items, spatial, and visual tasks suggested the STM/WM capacity of four items (Cowan, 2001; Vogel et al., 2001). Theoretically, the STM/WM capacity can be improved by increasing the theta cycle length, or by increasing the gamma frequencies, which increases the number of gamma waves that fit within a given theta cycle (Kamiński et al., 2011). Contrary to the expectations, Malenínská et al. (2021) found no association between the theta/gamma ratio and performance on digit span task (Malenínská et al., 2021).

Vosskuhl et al. (2015) artificially slowed theta frequency to increase the number of gamma waves per single theta cycle and found that the verbal STM capacity was improved compared with the sham stimulation (Vosskuhl et al., 2015). In another study, Wolinski et al. (2018) examined the effect of transcranial alternating current stimulation (tACS) administered at a slow theta (4 Hz), a fast theta frequency (7 Hz), and in a placebo condition over the right parietal cortex while performing visuospatial WM task. They found that tACS administered at 4 Hz had a positive effect on the WM performance, while tACS administered at 7 Hz had a detrimental effect (Wolinski et al., 2018).

In contrast to the first model which assumes that each gamma wave represents a single memory item, the second model assumes that each memory item is encoded by the entire gamma burst (Herman et al., 2013; Van Vugt et al., 2014). After a certain period of time, the memory items need to be refreshed through the new gamma bursts. This reactivation occurs after a few theta cycles, which could explain the limited WM capacity (Van Vugt et al., 2014). Based on this model, a slowing down theta cycle means that fewer memory items could be activated in a given period of time. Thus, one might expect a decrease rather than an increase in the WM capacity. However, the increase in the WM capacity reported by Vosskuhl et al. (2015) and Wolinski et al. (2018) could mean that the gain in memory fidelity due to the greater activation with the longer gamma burst displaces the memory decay resulting from the slowing down theta cycles (Vosskuhl et al., 2015; Wolinski et al., 2018). These two models can be used to predict the increase or decrease in the WM capacity at a given tACS frequency. Thus, based on these models one could design a brain stimulation protocol to boost WM (e.g., theta/gamma CFC tACS).

Differences in the WM capacity between individuals result in variations in several skills, such as attention, academic performance, and non-verbal reasoning ability (Gathercole et al., 2003; Kane et al., 2007). The reduced WM capacity is a common feature in many diseases, such as schizophrenia, stroke, traumatic brain injury, attention deficit hyperactivity disorder (ADHD), mild cognitive impairment (MCI), and AD (Baddeley et al., 1991; Gagnon and Belleville, 2011; Constantinidis and Klingberg, 2016). In addition, abnormal PACs have been associated with diseases, such as AD, epilepsy, mental disorders, and Parkinson's disease (Salimpour and Anderson, 2019). Taken together, this information sheds light on the possible role of CFC in WM and its potential role as a therapeutic target in such diseases. Improving the WM performance is a challenge and a hot topic in clinical practice, especially in patients with AD, MCI, etc., any progress in this area is beneficial. WM can be manipulated/modulated by various approaches, and non-invasive brain stimulation with an electric or magnetic field is one of them. Over the past two decades, there has been a long list of studies reporting the effects of frequency-tuned tACS, and transcranial magnetic stimulation (TMS) on WM (Jaušovec et al., 2014; Hoy et al., 2015, 2016; Chander et al., 2016; Feurra et al., 2016; Alekseichuk et al., 2017; Papazova et al., 2020). It is important to emphasize that there are different forms of tACS, some forms target specific individual frequency bands, such as theta, gamma, and beta (theta-tACS, gamma-tACS, beta-tACS, etc.), and CFC-tACS form; where tACS can modulate the interaction between the two frequency bands, such as theta and gamma (e.g., theta/gamma 6 Hz, 80 Hz peak-CFC-tACS, where gamma bursts at 80 Hz were nested into the peak of theta cycles at a frequency of 6 Hz, which is so-called peak-coupled tACS). Since the topic of this review is CFC of neuronal oscillations and WM in adult humans, the studies that have investigated the effect of non-invasive brain stimulation on CFC (e.g., CFC-tACS), as well as the studies that have investigated the association between CFC of neuronal oscillations and WM in adult humans, are thoroughly discussed in this review and listed in Table 1.

**Table 1:** Studies that investigated the association between the cross-frequency coupling (CFC) over different brain cortices and working memory (WM) performance.

**References**	**Recording method**	**Study details**	**Task(s)**	**Main findings**
	**Brain cortices involved**			
**Cross-frequency tACS between theta and gamma frequencies**				
Alekseichuk et al. (2016)	EEG Prefrontal cortex	✓ 47 participants ✓ Three experimental sets were used: subjects were instructed to complete tasks during sham stimulation (first set), continuous low-frequency theta stimulation (second set), and cross-frequency coupling tACS between theta and gamma frequencies (third set)	Two-back visual-spatial match-to-sample test	- The positive effect of continuous low-frequency entrainment on WM performance was abolished by synchronizing high gamma bursts with the troughs of theta cycles - Significant improvement in WM performance was found when high oscillation gamma bursts (80–100 Hz frequency range) were embedded in the peaks of theta cycles
**Theta/gamma coupling**				
Canolty et al. (2006)	iEEG Whole cortex	✓ 5 patients with epilepsy ✓ Investigated the relationship between TGC and behavioral outcomes	Behavioral tasks	- Theta/Gamma PAC distributed throughout the cortex and the strength of TGC increased in more cognitively demanding WM tasks - A significant effect was observed when gamma oscillations were detected in the trough of theta cycles
Axmacher et al. (2010)	iEEG Hippocampus	✓ 14 patients with epilepsy ✓ The relationship between TGC and WM maintenance was investigated ✓ Investigated the relationship between a relatively large number of WM - items and CFC	Sternberg paradigm	- WM maintenance was associated with TGC in the hippocampus - Modulation of beta/gamma amplitude and theta activity were associated with a relatively large number of WM items
Chaieb et al. (2015)	iEEG Hippocampus	✓ 14 patients with epilepsy ✓ Investigated phase-phase couplings in the hippocampus in presurgical patients with epilepsy using iEEG recordings	Serial Sternberg WM task	- Theta and beta/gamma phase-phase coupling in the hippocampus during retention of multiple WM items–Sternberg WM task–in pre-operative patients with epilepsy
Köster et al. (2014)	EEG Prefrontal-Parietal cortices	✓ 26 participants ✓ Examined and quantified cross-frequency coupling during the pictogram recognition task using EEG datasets	Pictorial recognition tasks	- Coupling between prefrontal theta phase and parietal gamma amplitude was enhanced for the retrieved items.
Holz et al. (2010)	EEG Partial-Occipital cortices	✓ 23 participants ✓ While participants completed a visuospatial delayed pattern matching task, EEG was recorded ✓ The relationship between TGC and WM performance was investigated	Delayed match-to-sample visual WM task	- Association between TGC and correctly identified items in delayed visual match-to-sample task WM was found
Griesmayr et al. (2010)	EEG Frontal Medline-distributed gamma activity	✓ 31 participants ✓ EEG power analysis was performed together with CFC analysis to test the relationship between TGC and behavioral	Verbal delayed match to sample task	- The coupling between frontal midline oscillatory theta and gamma activities correlated with temporal separation of memory item - Higher frontal midline theta power might be correlated with rehearsal processes during verbal delayed match to sample task
		performance in the verbal delayed matching task		
Friese et al. (2013)	EEG Frontal-Posterior cortices	✓ 26 participants ✓ EEG data were collected while participants performed the remember/know task.	Remember/know procedure	- Theta-gamma PAC in the frontal and posterior cortices was increased during the encoding process for visual stimuli - A decrease in prefrontal and occipital alpha-oscillatory activities was observed during successful encoding
Lee and Yang, 2014	EEG Frontal-Parietal cortices	✓ 9 participants ✓ Theta/gamma coupling during a visuo-spatial delayed-matching task was investigated	Visuo-Spatial delayed-matching task	- Significant correlation between correct responses and the synchronization index in the partial lobe
Park et al. (2011)	EEG Prefrontal-Parietal cortices	✓ 31 older adults ✓ Examined the association between TGC and behavioral outcomes using EEG	➢Spatial delayed match-to-sample ➢Delayed figure recall ➢Delayed verbal recall.	- TGC in parietal cortex was significantly associated with a high score on delayed figure recall task - The accuracy rate of the spatial delayed match-to-sample task was associated with TGC
Park et al. (2013)	EEG Frontal cortex	✓ 13 participants ✓ Examined TGC levels during simple vigilance and visuospatial WM tasks	2-back task vs. simple vigilance	- Theta/gamma coupling increased in the frontal area at 40 Hz during visuo-spatial WM task (2-back task)
Rajji et al. (2017)	EEG Frontal cortex	✓ 70 subjects ✓ N back task with 3 levels of difficulties (ordering information) ✓ EEG recording during n-back tasks ✓ Event related potential within each n-back condition	N-back task	- Theta/gamma coupling was significant for tasks requiring ordering information
Brooks et al. (2020)	EEG Prefrontal cortex	✓ 311 participants ✓ 3 groups (healthy control, mild cognitive impairment, major depressive disorder patients) ✓ Participants completed n-back and non-n-back tasks seven days apart ✓ The relationship between TGC and WM performance (using different tasks) was examined	➢N-back task ➢2 tasks require ordering information other than n-back task ➢3 tasks do not require ordering information	- Association between TGC and cognitive tasks that require order information (n-back task and non-n-back task) - These results were not influenced by clinical diagnosis - TGC was not associated with tasks that do not require ordering information - No association between diagnosis and TGC
Goodman et al. (2018)	EEG Frontal cortex	✓ 98 participants ✓ Alzheimer's dementia, mild cognitive impairment patients, and healthy control ✓ The association between theta/gamma CFC and WM in patients with mild cognitive impairment and Alzheimer's dementia patients was examined	N-back task	- TGC was the lowest in patients with Alzheimer's dementia, followed by mild cognitive impairment patients and finally healthy control
Tseng et al. (2019)	EEG Frontal and parietal regions	✓ 36 healthy participants ✓ Examined theta/gamma PAC during musical memory retrieval	Musical memory task	- Enhanced theta/gamma PAC during musical memory retrieval was observed in the frontal and parietal cortices
Graetz et al. (2019)	EEG Frontal and occipital	✓ 22 participants ✓ To examine the processes behind memorization of repeatedly presented stimuli	continuous item recognition task with up to five presentations per item	- At second presentation–theta amplitudes peaked - After second presentation—reduction in alpha suppression - After third presentation—in response time and a reduction in frontal theta/gamma PAC were observed
Fernández et al. (2021)	EEG Frontal and posterior cortices	✓ 25 participants ✓ Studying weather WM loads affects the interaction between brain oscillations in different brain cortices	Delayed-matching-to-sample with different WM loads	- PAC between theta phase and beta/gamma amplitude was modulated by WM load
**Alpha/gamma coupling**				
Voytek et al. (2010)	iEEG Whole cortex	✓ 2 patients with intractable epilepsy ✓ Alpha/theta and gamma coupling	Two non-visual (verb generation phoneme and word repetition, and phoneme and word target detection) and two visual tasks (lateralized visual target detection task and a visual context task)	- Theta and alpha phase modulated gamma amplitude - Over the anterior brain cortices, theta PAC is higher than alpha PAC, and alpha PAC over visual cortices was high during visual tasks
Pinal et al. (2015)	EEG Frontal and posterior cortices	✓ 20 young adults and 20 elderly ✓ Examined brain oscillatory activity in young and elderly during delayed match-to-sample task ✓ Alpha/gamma coupling	Delayed match-to-sample task	- In contrast to young participants, elderly participants maintained synchronization in the resting state network and lacked the ability to synchronize frontoparietal task-related network activities (alpha-gamma) during task performance
Park et al. (2016)	MEG Early visual cortex (occipital lobe)	✓ 23 participants ✓ Examined the dynamic interactions between alpha and gamma oscillations implicated in visual memory process ✓ ✓ Alpha/gamma coupling	Visual memory task (remember or not remember presented pictures)	- Decrease in alpha power and increase in alpha phase and gamma power during recall of images
Popov et al. (2018)	MEG Early visual cortex (dorsal and ventral visual streams)	✓ 83 participants ✓ Examined the relationship between brain oscillations (fast and slow frequencies) and behavioral outcomes ✓ Alpha/beta and gamma power/power coupling	N-back task	- Increased alpha/beta and gamma power/power interactions in early visual cortex (specifically dorsal and ventral visual streams) were found when n-back task demands were increased
**Other types of cross-frequency coupling**				
Daume et al. (2017a)	MEG Left inferior temporal cortex	✓ 27 participants ✓ Theta/alpha phases and beta amplitude PAC	Visual delayed match-to-sample task	- Interaction between theta/alpha phases and beta amplitude was demonstrated in left inferior temporal cortex using recorded MEG data. In this study, while participants completed visual delayed match-to-sample task, an increase in the power of beta and gamma oscillations and a decrease in the power of theta/alpha oscillations were observed in visual sensory areas during the delay period - The left inferior temporal cortex was connected to the prefrontal cortex via increased theta/alpha coupling
Siebenhühner et al. (2016)	Magneto-Electroencephalography Fronto-Parietal, dorsal attention, and visual areas	✓ 12 participants ✓ Theta and alpha–gamma coupling ✓ Alpha and beta-gamma coupling	Delayed match-to-sample visual WM task	- Enhanced couplings between theta and alpha–gamma and between alpha and beta-gamma bands during WM maintenance in fronto-parietal, dorsal attention and visual areas
Rodriguez-Larios and Alaerts (2019)	EEG Posterior frontotemporal area	✓ 51 participants ✓ Alpha-theta phase synchrony	Arithmetic task	- Increased alpha/theta phase synchrony was associated with improved arithmetic task outcomes
Dimitriadis et al. (2016)	EEG Parieto-Occipital and frontal cortices	✓ 16 young adults ✓ Investigated the functional coupling between WM sub-systems during arithmetic task performance ✓ Assessed correct and wrong responses	Arithmetic task with five cognitive demand levels	- PAC (frontal theta phase and parieto-occipital alpha amplitude) strength decreased with increasing difficulty of both correct and incorrect trials
Daume et al. (2017b)	MEG Left inferior temporal cortex and medial temporal lobe	✓ 29 participants ✓ Examined whether increased low-frequency phase synchronization between sensory areas is associated with audio-visual WM compared to visual WM ✓ Theta/beta cross-frequency coupling	Audio-Visual delayed match-to-sample task	- Increased theta/beta PAC during the WM delay period was observed in the medial temporal lobe and phase synchronization (theta rage) was stronger than that of the lateral prefrontal cortex in audio-visual WM compared to visual WM - Increased phase synchronization between medial temporal lobe and temporo-occipital areas in beta-band frequency

*iEEG, intracranial electroencephalography; TGC, theta/gamma coupling; PAC, phase- amplitude coupling; WM, working memory; CFC, cross-frequency coupling; EEG, electroencephalography; MEG, magnetoencephalography; tACS, transcranial alternating current stimulation*.

#### Theta-Gamma Neural Code and WM

The relationship between the brain oscillations and WM capacity has been investigated in the different brain regions (parietal, frontal, occipital regions, hippocampus…etc.), and several studies have focused on the association between CFC, particularly theta-gamma coupling (TGC), and WM performance (Schack et al., 2002; Demiralp et al., 2007; Mizuhara and Yamaguchi, 2011; Bahramisharif et al., 2018; Biel et al., 2021). Theta/gamma PAC is found in the hippocampus and other brain structures (Maris et al., 2011; Belluscio et al., 2012; van der Meij et al., 2012; Colgin, 2015) and provides a code for representing and maintaining the multiple WM items-theta/gamma neural codes (Axmacher et al., 2010; Lisman and Jensen, 2013). The theta/gamma neural code hypothesis posits that the conserved memory items are registered via theta-nested gamma cycles in the sensory regions. Accordingly, the theta-gamma neural code coordinates communication between the different brain cortices during memory and sensory processes (Lisman and Jensen, 2013) and is specifically correlated to the WM requirements (Park et al., 2013).

Several studies have used intracranial EEG data in patients with epilepsy along with the behavioral outcomes to demonstrate the association between theta/gamma CFC across different brain regions and WM performance (Canolty et al., 2006; Rizzuto et al., 2006; Axmacher et al., 2010; Freunberger et al., 2011; Chuderski, 2016; Chai et al., 2018). The results of these studies can be summarized as follows: theta/gamma PAC distributed across the cortex and the strength of TGC increased with more cognitively demanding WM tasks. Moreover, a significant effect was observed when the gamma oscillations were detected in the trough of theta cycles (Canolty et al., 2006). In general, the theta troughs and peaks have different functions: WM retrieval occurs during the peaks, whereas WM encoding occurs during the troughs (Rizzuto et al., 2006). Axmacher et al. found that the WM maintenance is associated with the TGC in the hippocampus and the modulation of beta/gamma amplitude and theta activity were associated with a relatively large number of WM items (Axmacher et al., 2010). On the other hand, Chaieb et al. observed theta and beta/gamma phase-phase coupling in the hippocampus during the maintenance of multiple WM items (Sternberg WM task) in pre-surgical patients with epilepsy (Chaieb et al., 2015). All these studies are summarized in Table 1.

Cross-frequency coupling supports the organization of brain rhythms and is present during a range of cognitive functions. However, little is known about whether and how long-range CFC across the distant brain regions subserves WM. Here we report that theta–slow gamma coupling between the hippocampus and medial prefrontal cortex (mPFC) is augmented in a genetic mouse model of cognitive dysfunction. This increased CFC is observed specifically when the mice successfully perform a spatial WM task. In wild-type mice, increasing task difficulty by introducing a long delay or by optogenetically interfering with encoding, also increases the theta–gamma coupling during correct trials. Finally, the epochs of high hippocampal theta–prefrontal slow gamma coupling are associated with the increased synchronization of neurons within the mPFC. These findings suggest that the enhancement of theta–slow gamma coupling reflects a compensatory mechanism to maintain spatial WM performance in the setting of increased difficulty. The association between WM and theta-gamma PAC in the frontal, parietal, occipital, and posterior cortices has been reported in several studies using the different tasks (pictorial recognition tasks, delayed match-to-sample visual WM task, verbal delayed match to sample task, delayed figure recall, n-back task, etc.) to demonstrate the association of interest by using the EEG recordings (Griesmayr et al., 2010; Holz et al., 2010; Park et al., 2011; Friese et al., 2013; Köster et al., 2014; Lee and Yang, 2014; Graetz et al., 2019; Tseng et al., 2019; Fernández et al., 2021). The lowest level of TGC coupling was found in the patients with Alzheimer's dementia, followed by the patients with MCI and finally healthy controls (Goodman et al., 2018). All the studies showed modulations in the theta-gamma CFC related to correctly identified/retrieved items. Besides that, a decrease in the prefrontal and occipital alpha oscillatory activities was observed by Friese et al. (2013). The details of the studies are summarized in Table 1. The combined effect of WM training and transcranial direct current stimulation (tDCS) on the behavioral changes was investigated using the EEG features and cognitive task scores, and it was found that an increase in PAC between the prefrontal theta oscillations and temporo-parietal gamma oscillations was associated with the improvement in behavioral task scores, and more prominent improvement was found when the gamma waves coincided near the theta peaks (Jones et al., 2020).

The theta/gamma PAC strength is influenced by the oscillatory activities of other frequency bands; alpha amplitude influenced TGC in the two intracranial electroencephalography (iEEG) experiments that used different WM tasks (Leszczyński et al., 2015). Sauseng et al. found that repetitive TMS at alpha frequency suppressed distracting information and could influence the STM capacity (Sauseng et al., 2009). On the other hand, delta-alpha coupling influenced TGC and supported switching between the WM modes in the hippocampal region (Leszczyński et al., 2015).

#### Alpha-Gamma Coupling and WM

The default mode network (DMN) arises from the medial prefrontal cortex, the posterior cingulate cortex, and the inferior parietal cortex (Buckner et al., 2008). It is associated with episodic memory and self-referential thinking (Buckner et al., 2008; Knyazev et al., 2011; Weiler et al., 2014). The activity of DMN is higher during the resting state as compared with the task-performing state (Buckner et al., 2008). Indeed, an association between the DMN and WM networks has been suggested where DMN nodes could be activated during the memory phases (Hahn et al., 2007; Vilberg and Rugg, 2008; Daselaar et al., 2009). Moreover, the activity of DMN was mediated by alpha and beta oscillations, and the connectivity between some DMN parts is correlated to the alpha oscillatory activities (Hacker et al., 2017; Tang et al., 2017). A decrease in DMN functional connectivity and alpha power has been detected in patients with AD (Jeong, 2004; Zhang et al., 2009). In contrast to the young adults, old adults maintained synchronization in the resting state network and lacked the ability to synchronize the frontoparietal task-related network activities during the task performance (Pinal et al., 2015).

The electrophysiological studies have shown that when the content of WM changes from multiple items to distinct visual or spatial information, the oscillatory theta activities are replaced by alpha ones (Fries et al., 2001; Sauseng et al., 2005). Moreover, the studies examining the brain oscillations during the WM delay period found that the theta oscillatory activities occurred predominantly in the tasks that required sequential multiple WM item coding, while the alpha-oscillatory activities occurred in the tasks that required retention of visual or spatial information presented simultaneously (Roux and Uhlhaas, 2014). CFC between the parieto-occipital alpha activity and topographically distributed gamma activity is involved in prioritizing different visual representations in WM and deficits in the prefrontal cortex disrupt this process (Davoudi et al., 2021).

Alpha/gamma coupling has been demonstrated during visual WM maintenance in patients with epilepsy (Voytek et al., 2010). Park et al. used MEG data from healthy participants who were asked to recall the displayed images and found a reduction in the alpha power and an enhancement of alpha/gamma PAC during the process of recalling the images (Park et al., 2016). Moreover, alpha/beta and gamma power in the early visual cortex (in the dorsal and ventral visual streams) were modulated in response to the demands of the n-back task (Popov et al., 2018). The details of the studies are summarized in Table 1. Presumably, the theta and alpha activities are localized across different EEG recording sites. This assumption is supported by electrocorticography recordings, which showed that the alpha/gamma coupling was detected in the parietal–occipital cortices during the tasks requiring visual information processing, whereas theta/gamma PAC was predominantly observed in the frontotemporal regions during non-visual tasks (Voytek et al., 2010).

#### Other Types of CFC and WM

In principle, PAC can be generated in many ways; one source of the low-frequency band can be coupled to one or more sources of high-frequency bands. Thus, PAC is a general phenomenon and is not restricted to the theta/gamma frequencies. WM affects PAC in different ways; as WM could increase the PAC strength in some patterns and decrease it in others (Maris et al., 2011). CFC between the neural oscillations other than theta/gamma and alpha/gamma has been demonstrated during the WM maintenance as follows: (1) enhanced coupling between the theta/alpha phases and amplitude of beta has been demonstrated in temporal cortex during the visual delayed match-to-sample task (Daume et al., 2017a), (2) improved beta-theta PAC in the medial temporal lobe during the WM delay period (audio-visual delayed match to sample task) and enhanced phase synchronization between the medial temporal lobe and temporo-occipital areas in the beta band frequency range (Daume et al., 2017b), (3) improved the couplings between theta and alpha–gamma and between alpha and beta-gamma bands during the WM maintenance (delayed match-to-sample visual WM task) in the frontoparietal, dorsal, and visual areas (Siebenhühner et al., 2016), (4) increased alpha/theta phase synchrony was associated with the improved arithmetic task outcomes in the posterior and frontoparietal regions (Rodriguez-Larios and Alaerts, 2019), (5) the PAC (frontal theta phase and parieto-occipital alpha amplitude) strength decreased with increasing difficulty of both the correct and incorrect responses to the arithmetic tasks (Dimitriadis et al., 2016). The details of the studies are summarized in Table 1.

#### Frequency-Tuned Non-invasive Stimulation CFC and WM

The non-invasive brain stimulation methods are a potent way to study the causal relationship between brain activities and behavioral outcomes. These methods could causally modulate the behavior using electric or magnetic fields (Walsh and Cowey, 2000; Miniussi and Ruzzoli, 2013). Several studies have investigated the effect of frequency-tuned tACS and TMS on the WM performance with mixed results (Jaušovec et al., 2014; Hoy et al., 2015; Chander et al., 2016; Alekseichuk et al., 2017; Kuhnke et al., 2017; Sreeraj et al., 2017; Borghini et al., 2018; Wolinski et al., 2018; Jones et al., 2019; Beynel et al., 2020; Kehler et al., 2020; Papazova et al., 2020) and others. In the context of WM and CFC, a study was conducted to demonstrate the direct causality between the CFC and WM performance, the interaction between theta and gamma bands in the prefrontal cortex was externally modulated by CFC tACS protocol (Alekseichuk et al., 2016). The volunteers were instructed to perform the tasks during the stimulation and were assigned into three groups: sham stimulation (first group), continuous single-frequency theta stimulation (second group), and CFC tACS between theta and gamma frequencies (third group). Based on the behavioral and EEG data, the positive effect of continuous low-frequency entrainment on the WM performance was canceled out by the synchronization of high gamma bursts with the troughs of theta cycles (Alekseichuk et al., 2016). In contrast, a significant improvement in the WM performance was found when high oscillations gamma bursts (80–100-Hz frequency range) were embedded in the peaks of theta cycles (Alekseichuk et al., 2016).

## Discussion

Overall, this review reports the association between the CFC and WM performance, almost all the studies showed a relationship between the CFC in different brain regions and WM performance, especially WM maintenance. Theta/gamma PAC is the most commonly reported CFC model in this context. The causal role of oscillations in memory processes can be realized by modulating endogenous oscillations and precisely determining the behavioral effects of such modulation. Entrainment of oscillations can be achieved by various methods and non-invasive brain stimulation is one of them. Non-invasive brain stimulation is a robust tool to establish a causal relationship between the neuronal oscillations at the mesoscopic scale and their role in cognition (Romei et al., 2011). The disadvantages of non-invasive brain stimulation methods are (1) high inter-subject variability (López-Alonso et al., 2014); (2) weak and inconsistent results of different studies (Ziemann and Siebner, 2015). (30) inconsistent long-lasting aftereffects (Veniero et al., 2015). Frequency-tuned non-invasive stimulation is a recent approach in neuroscience, in which the frequency of transcranially applied electromagnetic currents is matched to the ongoing oscillatory components with the aim of altering the behavior (Veniero et al., 2015; Albouy et al., 2018). WM can be manipulated/modulated by various approaches, and frequency-tuned non-invasive brain stimulation with an electric or magnetic field is one of them. Over the past two decades, there has been a long list of studies reporting the effects of tACS and TMS on WM (Jaušovec and Jaušovec, 2014; Hoy et al., 2015, 2016; Alekseichuk et al., 2016; Chander et al., 2016; Feurra et al., 2016; Kuhnke et al., 2017; Jones et al., 2019; Beynel et al., 2020; Kehler et al., 2020) and others. In general, theta-tACS improved the WM outcomes in the majority of studies, whereas the effect of gamma-tACS, alpha-tACS, etc., on WM appears inconsistent. This is partly due to the heterogeneity of the experimental setups and stimulation sites (frontal, parietal, and occipital) used in these studies. The CFC-tACS protocols have been investigated in several domains (learning, WM, and verbal-long term memory) (Alekseichuk et al., 2016; Lara et al., 2018; Turi et al., 2020; Riddle et al., 2021) and have recently gained popularity among the researchers. In principle, the peak-coupled tACS (gamma bursts nested into theta peaks) protocols mimic the endogenous theta-gamma CFC phase specificity needed for cognitive control (Smith et al., 2015). Thus, one might expect that the peak-coupled theta-gamma tACS would improve cognitive functions compared with the sham stimulation. In the context of WM, the causal role of CFC was demonstrated by Alekseichuk et al. (2016) in which exogenously applied theta and gamma CFC tACS were adjusted to the intrinsic continuous theta and repetitive gamma waves in the prefrontal cortices of healthy participants. Interestingly, theta-gamma tACS boosted working memory more than theta-tACS alone, and the effect was more pronounced when the gamma bursts (in the range of 80–100 Hz) were over the peak of the theta cycles (peak-coupled tACS). Alekseichuk et al. (2016) and Turi et al. (2020) investigated the effect of theta/gamma CFC tACS protocols aimed at the stimulating frontal and cingulate cortices on Go/NoGo monetary reward-based and punishment-based instrumental learning task outcomes. They used different theta/gamma CFC tACS protocols, in contrast to the results of (Alekseichuk et al., 2016) This study showed no consistent reinforcement effect of peak-coupled tACS, whereas trough-coupled tACS (gamma bursts were nested into theta troughs) impaired cognitive control (Alekseichuk et al., 2016; Turi et al., 2020). Furthermore, Riddle et al. found that the delta/beta peak-coupled tACS (beta oscillations in the prefrontal cortex were nested into the peak of delta cycles in the prefrontal cortex) and theta/gamma peak-coupled tACS (gamma bursts in the parietal-occipital were nested into the peak of theta cycles in the prefrontal cortex) modulated the cognitive task outcomes (Riddle et al., 2021). Additionally, Lara et al. examined the effect of theta/gamma CFC tACS during the verbal long-term memory encoding, the results of this study were consistent with those of Lara et al. (2018). Thus, the effect of CFC-tACS on the brain function outcomes could vary depending on the domain tested (WM, learning, long-term memory, etc.), brain regions activated, and experimental setups used. In general, the WM performance is sensitive to the phase and rhythm of externally applied tACS (Alekseichuk et al., 2016), as well as to the area of stimulation (brain cortices) and the experimental setups. In the context of WM and CFC, Alekseichuk et al. (2016) study opens the way for the promising research on frequency tuned non-invasive brain stimulation protocols to modulate the CFC activities instead of only modulating the narrow banded oscillatory activities. Furthermore, future studies should investigate the effect of CFC-tACS on WM in the healthy participants and in the patients with WM deficits as the two groups differ substantially in their susceptibility to tACS effect (Hoy et al., 2015, 2016; Dallmer-Zerbe et al., 2020).

In summary, the association between CFC and WM has been demonstrated in many studies. The effect of CFC tACS on the WM outcomes needs to be comprehensively studied using different brain regions involved in the WM processing and different experimental setups to achieve a consistent effect that is associated with the acceptable behavioral improvement and minimal tACS-induced side effects. Innovative approaches to validate the tACS effects in realistic settings are needed before CFC-tACS can modulate everyday cognitive performance and be used as a promising therapeutic tool.

## Author Contributions

MA conducted the literature search and the summaries of previous published studies. MA and WA wrote the first and the final version of the manuscript. EK contributed to the progress of the manuscript and provided guidance with expert perspective. All authors contributed and approved the final version of the manuscript.

## Conflict of Interest

The authors declare that the research was conducted in the absence of any commercial or financial relationships that could be construed as a potential conflict of interest.

## Publisher's Note

All claims expressed in this article are solely those of the authors and do not necessarily represent those of their affiliated organizations, or those of the publisher, the editors and the reviewers. Any product that may be evaluated in this article, or claim that may be made by its manufacturer, is not guaranteed or endorsed by the publisher.

## Abbreviations

WM: working memory
ADHD: attention deficit hyperactivity disorder
MCI: mild cognitive impairment
AD: Alzheimer's disease
CFC: cross frequency coupling
PAC: phase amplitude coupling
EEG: electroencephalography
MEG: magnetoencephalography
iEEG: intracranial electroencephalography
STM: short term memory
tACS: transcranial alternating current stimulation
tDCS: transcranial direct current stimulation
TMS: transcranial magnetic stimulation
TGC: theta gamma coupling
DMN: default mode network.

## References

[B1] Aben B. Stapert S. Blokland A. (2012). About the distinction between working memory and short-term memory. Front. Psychol. 3:301. 10.3389/fpsyg.2012.00301 22936922 PMC3425965

[B2] Ackerman P. L. Beier M. E. Boyle M. O. (2005). Working memory and intelligence: the same or different constructs? Psychol. Bull. 131, 30–60. 10.1037/0033-2909.131.1.30 15631550

[B3] Albouy P. Baillet S. Zatorre R. J. (2018). Driving working memory with frequency-tuned noninvasive brain stimulation. Ann. N. Y. Acad. Sci. 1423, 126–137. 10.1111/nyas.13664 29707781

[B4] Alegre M. (2016). Cross-frequency coupling in the pathophysiology of Parkinson's disease. Clin. Neurophysiol. 127:e29. 10.1016/j.clinph.2015.11.087

[B5] Alekseichuk I. Pabel S. C. Antal A. Paulus W. (2017). Intrahemispheric theta rhythm desynchronization impairs working memory. Restor. Neurol. Neurosci. 35, 147–158. 10.3233/RNN-160714 28059806

[B6] Alekseichuk I. Turi Z. Amador de Lara G. Antal A. Paulus W. (2016). Spatial working memory in humans depends on theta and high gamma synchronization in the prefrontal cortex. Curr. Biol. 26, 1513–1521. 10.1016/j.cub.2016.04.035 27238283

[B7] Allen E. A. Liu J. Kiehl K. A. Gelernter J. Pearlson G. D. Perrone-Bizzozero N. I.. (2011). Components of cross-frequency modulation in health and disease. Front. Syst. Neurosci. 5:59. 10.3389/fnsys.2011.00059 21808609 PMC3139214

[B8] Andersen L. M. Jerbi K. Dalal S. S. (2020). Can EEG and MEG detect signals from the human cerebellum? Neuroimage 215:116817. 10.1016/j.neuroimage.2020.116817 32278092 PMC7306153

[B9] Axmacher N. Henseler M. M. Jensen O. Weinreich I. Elger C. E. Fell J. (2010). Cross-frequency coupling supports multi-item working memory in the human hippocampus. Proc. Natl. Acad. Sci. U.S.A. 107, 3228–3233. 10.1073/pnas.0911531107 20133762 PMC2840289

[B10] Baddeley A. (1992). Working memory. Science 255, 556–559. 10.1126/science.1736359 1736359

[B11] Baddeley A. D. Bressi S. Della Sala S. Logie R. Spinnler H. (1991). The decline of working memory in alzheimer's disease: a longitudinal study. Brain 114, 2521–2542. 10.1093/brain/114.6.2521 1782529

[B12] Bahramisharif A. Jensen O. Jacobs J. Lisman J. (2018). Serial representation of items during working memory maintenance at letter-selective cortical sites. PLoS Biol. 16:e2003805. 10.1371/journal.pbio.2003805 30110320 PMC6093599

[B13] Başar E. (2013). Brain oscillations in neuropsychiatric disease. Dialogues Clin. Neurosci. 15, 291–300. 10.31887/DCNS.2013.15.3/ebasar 24174901 PMC3811101

[B14] Belluscio M. A. Mizuseki K. Schmidt R. Kempter R. Buzsáki G. (2012). Cross-frequency phase-phase coupling between theta and gamma oscillations in the hippocampus. J. Neurosci. 32, 423–435. 10.1523/JNEUROSCI.4122-11.2012 22238079 PMC3293373

[B15] Beynel L. Davis S. W. Crowell C. A. Dannhauer M. Lim W. Palmer H.. (2020). Site-specific effects of online rTMS during a working memory task in healthy older adults. Brain Sci. 10:255. 10.3390/BRAINSCI10050255 32349366 PMC7287855

[B16] Biel A. L. Minarik T. Sauseng P. (2021). EEG cross-frequency phase synchronization as an index of memory matching in visual search. Neuroimage 235:117971. 10.1016/j.neuroimage.2021.117971 33839263

[B17] Borghini G. Candini M. Filannino C. Hussain M. Walsh V. Romei V.. (2018). Alpha oscillations are causally linked to inhibitory abilities in ageing. J. Neurosci. 38, 4418–4429. 10.1523/JNEUROSCI.1285-17.2018 29615485 PMC6596011

[B18] Botvinick M. Watanabe T. (2007). From numerosity to ordinal rank: A gain-field model of serial order representation in cortical working memory. J. Neurosci. 27, 8636–8642. 10.1523/JNEUROSCI.2110-07.2007 17687041 PMC6672950

[B19] Brooks H. Goodman M. S. Bowie C. R. Zomorrodi R. Blumberger D. M. Butters M. A.. (2020). Theta–gamma coupling and ordering information: a stable brain–behavior relationship across cognitive tasks and clinical conditions. Neuropsychopharmacology 45, 2038–2047. 10.1038/s41386-020-0759-z 32682324 PMC7547084

[B20] Bruns A. Eckhorn R. (2004). Task-related coupling from high- to low-frequency signals among visual cortical areas in human subdural recordings. Int. J. Psychophysiol. 51, 97–116. 10.1016/j.ijpsycho.2003.07.001 14693360

[B21] Buckner R. L. Andrews-Hanna J. R. Schacter D. L. (2008). The brain's default network: anatomy, function, and relevance to disease. Ann. N. Y. Acad. Sci. 1124, 1–38. 10.1196/annals.1440.011 18400922

[B22] Buschman T. J. Miller E. K. (2007). Top-down versus bottom-up control of attention in the prefrontal and posterior parietal cortices. Science 315, 1860–1864. 10.1126/science.1138071 17395832

[B23] Buzsáki G. Draguhn A. (2004). Neuronal olscillations in cortical networks. Science 304, 1926–1929. 10.1126/science.1099745 15218136

[B24] Calderone D. J. Lakatos P. Butler P. D. Castellanos F. X. (2014). Entrainment of neural oscillations as a modifiable substrate of attention. Trends Cogn. Sci. 18, 300–309. 10.1016/j.tics.2014.02.005 24630166 PMC4037370

[B25] Canolty R. T. Edwards E. Dalal S. S. Soltani M. Nagarajan S. S. Kirsch H. E.. (2006). High gamma power is phase-locked to theta oscillations in human neocortex. Science 313, 1626–1628. 10.1126/science.1128115 16973878 PMC2628289

[B26] Canolty R. T. Knight R. T. (2010). The functional role of cross-frequency coupling. Trends Cogn. Sci. 14, 506–515. 10.1016/j.tics.2010.09.001 20932795 PMC3359652

[B27] Chai W. J. Abd Hamid A. I. Abdullah J. M. (2018). Working memory from the psychological and neurosciences perspectives: a review. Front. Psychol. 9, 401. 10.3389/fpsyg.2018.00401 29636715 PMC5881171

[B28] Chaieb L. Leszczynski M. Axmacher N. Höhne M. Elger C. E. Fell J. (2015). Theta-gamma phase-phase coupling during working memory maintenance in the human hippocampus. Cogn. Neurosci. 6, 149–157. 10.1080/17588928.2015.1058254 26101947

[B29] Chander B. S. Witkowski M. Braun C. Robinson S. E. Born J. Cohen L. G.. (2016). tACS phase locking of frontal midline theta oscillations disrupts working memory performance. Front. Cell. Neurosci. 10:120. 10.3389/fncel.2016.00120 27199669 PMC4858529

[B30] Chuderski A. (2016). Fluid intelligence and the cross-frequency coupling of neuronal oscillations. Span. J. Psychol. 19:E91. 10.1017/sjp.2016.86 27919297

[B31] Cohen M. X. (2014). A neural microcircuit for cognitive conflict detection and signaling. Trends Neurosci. 37, 480–490. 10.1016/j.tins.2014.06.004 25034536

[B32] Colgin L. L. (2015). Theta-gamma coupling in the entorhinal-hippocampal system. Curr. Opin. Neurobiol. 31, 45–50. 10.1016/j.conb.2014.08.001 25168855 PMC4340819

[B33] Colom R. Shih P. C. Flores-Mendoza C. Quiroga M. Á. (2006). The real relationship between short-term memory and working memory. Memory 14, 804–813. 10.1080/09658210600680020 16938693

[B34] Constantinidis C. Klingberg T. (2016). The neuroscience of working memory capacity and training. Nat. Rev. Neurosci. 17, 438–449. 10.1038/nrn.2016.43 27225070

[B35] Conway A. R. A. Kane M. J. Bunting M. F. Hambrick D. Z. Wilhelm O. Engle R. W. (2005). Working memory span tasks: A methodological review and user's guide. Psychon. Bull. Rev. 12, 769–786. 10.3758/BF03196772 16523997

[B36] Cowan N. (2001). The magical number 4 in short-term memory: a reconsideration of mental storage capacity. Behav. Brain Sci. 24, 87–114. 10.1017/S0140525X01003922 11515286

[B37] Cowan N. (2014). Working memory underpins cognitive development, learning, and education. Educ. Psychol. Rev. 26, 197–223. 10.1007/s10648-013-9246-y 25346585 PMC4207727

[B38] Cowan N. (2017). The many faces of working memory and short-term storage. Psychon. Bull. Rev. 24, 1158–1170. 10.3758/s13423-016-1191-6 27896630

[B39] Dallmer-Zerbe I. Popp F. Lam A. P. Philipsen A. Herrmann C. S. (2020). Transcranial alternating current stimulation (tACS) as a tool to modulate p300 amplitude in attention deficit hyperactivity disorder (ADHD): preliminary findings. Brain Topogr. 33, 191–207. 10.1007/s10548-020-00752-x 31974733 PMC7066286

[B40] Daselaar S. M. Prince S. E. Dennis N. A. Hayes S. M. Kim H. Cabeza R. (2009). Posterior midline and ventral parietal activity is associated with retrieval success and encoding failure. Front. Hum. Neurosci. 3:2009. 10.3389/neuro.09.013.2009 19680466 PMC2726033

[B41] Daume J. Graetz S. Gruber T. Engel A. K. Friese U. (2017b). Cognitive control during audiovisual working memory engages frontotemporal theta-band interactions. Sci. Rep. 7:12585. 10.1038/s41598-017-12511-3 28974716 PMC5626716

[B42] Daume J. Gruber T. Engel A. K. Friese U. (2017a). Phase-amplitude coupling and long-range phase synchronization reveal frontotemporal interactions during visual working memory. J. Neurosci. 37, 313–322. 10.1523/JNEUROSCI.2130-16.2017 28077711 PMC6596581

[B43] Davoudi S. Dezfouli M. P. Knight R. T. Daliri M. R. Johnson E. L. (2021). Prefrontal lesions disrupt posterior alpha–gamma coordination of visual working memory representations. J. Cogn. Neurosci. 33, 1798–1810. 10.1162/jocn_a_01715 34375418 PMC8428813

[B44] De Hemptinne C. Ryapolova-Webb E. S. Air E. L. Garcia P. A. Miller K. J. Ojemann J. G.. (2013). Exaggerated phase-amplitude coupling in the primary motor cortex in Parkinson disease. Proc. Natl. Acad. Sci. U.S.A. 110, 4780–4785. 10.1073/pnas.1214546110 23471992 PMC3606991

[B45] Demiralp T. Bayraktaroglu Z. Lenz D. Junge S. Busch N. A. Maess B.. (2007). Gamma amplitudes are coupled to theta phase in human EEG during visual perception. Int. J. Psychophysiol. 64, 24–30. 10.1016/j.ijpsycho.2006.07.005 16956685

[B46] DeStefano D. LeFevre J. (2010). The role of working memory in mental arithmetic. Cogn. Psychol. 16, 353–386. 10.1080/0954144024400032832295484

[B47] Dimitriadis S. I. Sun Y. Thakor N. V. Bezerianos A. (2016). Causal Interactions between frontalθ – parieto-occipitalα2 predict performance on a mental arithmetic task. Front. Hum. Neurosci. 10:454. 10.3389/fnhum.2016.00454 27683547 PMC5022172

[B48] Düzel E. Penny W. D. Burgess N. (2010). Brain oscillations and memory. Curr. Opin. Neurobiol. 20, 143–149. 10.1016/j.conb.2010.01.004 20181475

[B49] Edin F. Klingberg T. Johansson P. McNab F. Tegnér J. Compte A. (2009). Mechanism for top-down control of working memory capacity. Proc. Natl. Acad. Sci. U.S.A. 106, 6802–6807. 10.1073/pnas.0901894106 19339493 PMC2672558

[B50] Egner T. Gruzelier J. H. (2004). EEG Biofeedback of low beta band components: frequency-specific effects on variables of attention and event-related brain potentials. Clin. Neurophysiol. 115, 131–139. 10.1016/S1388-2457(03)00353-5 14706480

[B51] Engel A. K. Fries P. Singer W. (2001). Dynamic predictions: Oscillations and synchrony in top–down processing. Nat. Rev. Neurosci. 2, 704–716. 10.1038/35094565 11584308

[B52] Engle R. W. Tuholski S. W. Laughlin J. E. Conway A. R. A. (1999). Working memory, short-term memory, and general fluid intelligence: a latent-variable approach. J. Exp. Psychol. Gen. 128, 309–331. 10.1037/0096-3445.128.3.309 10513398

[B53] Fernández A. Pinal D. Díaz F. Zurrón M. (2021). Working memory load modulates oscillatory activity and the distribution of fast frequencies across frontal theta phase during working memory maintenance. Neurobiol. Learn. Mem. 183, 107476. 10.1016/j.nlm.2021.107476 34087476

[B54] Feurra M. Galli G. Pavone E. F. Rossi A. Rossi S. (2016). Frequency-specific insight into short-term memory capacity. J. Neurophysiol. 116, 153–158. 10.1152/jn.01080.2015 27121583 PMC4961742

[B55] Fougnie D. Zughni S. Godwin D. Marois R. (2015). Working memory storage is intrinsically domain specific. J. Exp. Psychol. Gen. 144, 30–47. 10.1037/a0038211 25384164

[B56] Freunberger R. Werkle-Bergner M. Griesmayr B. Lindenberger U. Klimesch W. (2011). Brain oscillatory correlates of working memory constraints. Brain Res. 1375, 93–102. 10.1016/j.brainres.2010.12.048 21172316

[B57] Fries P. (2009). Neuronal gamma-band synchronization as a fundamental process in cortical computation. Annu. Rev. Neurosci. 32, 209–224. 10.1146/annurev.neuro.051508.135603 19400723

[B58] Fries P. (2015). Rhythms for cognition: communication through coherence. Neuron 88, 220–235. 10.1016/j.neuron.2015.09.034 26447583 PMC4605134

[B59] Fries P. Nikolić D. Singer W. (2007). The gamma cycle. Trends Neurosci. 30, 309–316. 10.1016/j.tins.2007.05.005 17555828

[B60] Fries P. Reynolds J. H. Rorie A. E. Desimone R. (2001). Modulation of oscillatory neuronal synchronization by selective visual attention. Science 291, 1560–1563. 10.1126/science.1055465 11222864

[B61] Friese U. Köster M. Hassler U. Martens U. Trujillo-Barreto N. Gruber T. (2013). Successful memory encoding is associated with increased cross-frequency coupling between frontal theta and posterior gamma oscillations in human scalp-recorded EEG. Neuroimage 66, 642–647. 10.1016/j.neuroimage.2012.11.002 23142278

[B62] Gagnon L. G. Belleville S. (2011). Working memory in mild cognitive impairment and Alzheimer's disease: contribution of forgetting and predictive value of complex span tasks. Neuropsychology 25, 226–236. 10.1037/a0020919 21090897

[B63] Gathercole S. Brown L. Pickering S. (2003). Working memory assessments at school entry as longitudinal predictors of National Curriculum attainment levels. Educ. Child Psychol. 20, 109–122. Available at: https://research-information.bris.ac.uk/en/publications/working-memory-assessments-at-school-entry-as-longitudinal-predic (accessed February 5, 2021).

[B64] Gathercole S. E. Alloway T. P. (2006). Practitioner review: Short-term and working memory impairments in neurodevelopmental disorders: diagnosis and remedial support. J. Child Psychol. Psychiatry Allied Discip. 47, 4–15. 10.1111/j.1469-7610.2005.01446.x 16405635

[B65] Gignac G. E. (2015). The magical numbers 7 and 4 are resistant to the flynn effect: no evidence for increases in forward or backward recall across 85 years of data. Intelligence 48, 85–95. 10.1016/j.intell.2014.11.001

[B66] Goodman M. S. Kumar S. Zomorrodi R. Ghazala Z. Cheam A. S. M. Barr M. S.. (2018). Theta-Gamma coupling and working memory in Alzheimer's dementia and mild cognitive impairment. Front. Aging Neurosci. 10:101. 10.3389/fnagi.2018.00101 29713274 PMC5911490

[B67] Graetz S. Daume J. Friese U. Gruber T. (2019). Alterations in oscillatory cortical activity indicate changes in mnemonic processing during continuous item recognition. Exp. Brain Res. 237, 573–583. 10.1007/s00221-018-5439-4 30488235

[B68] Griesmayr B. Gruber W. R. Klimesch W. Sauseng P. (2010). Human frontal midline theta and its synchronization to gamma during a verbal delayed match to sample task. Neurobiol. Learn. Mem. 93, 208–215. 10.1016/j.nlm.2009.09.013 19808098

[B69] Hacker C. D. Snyder A. Z. Pahwa M. Corbetta M. Leuthardt E. C. (2017). Frequency-specific electrophysiologic correlates of resting state fMRI networks. Neuroimage 149, 446–457. 10.1016/j.neuroimage.2017.01.054 28159686 PMC5745814

[B70] Hahn B. Ross T. J. Stein E. A. (2007). Cingulate activation increases dynamically with response speed under stimulus unpredictability. Cereb. Cortex 17, 1664–1671. 10.1093/cercor/bhl075 16963517 PMC2693262

[B71] Hanslmayr S. Axmacher N. Inman C. S. (2019). Modulating human memory via entrainment of brain oscillations. Trends Neurosci. 42, 485–499. 10.1016/j.tins.2019.04.004 31178076

[B72] Helfrich R. F. Herrmann C. S. Engel A. K. Schneider T. R. (2016). Different coupling modes mediate cortical cross-frequency interactions. Neuroimage 140, 76–82. 10.1016/j.neuroimage.2015.11.035 26608244

[B73] Herman P. A. Lundqvist M. Lansner A. (2013). Nested theta to gamma oscillations and precise spatiotemporal firing during memory retrieval in a simulated attractor network. Brain Res. 1536, 68–87. 10.1016/j.brainres.2013.08.002 23939226

[B74] Herrmann C. S. Strüber D. Helfrich R. F. Engel A. K. (2016). EEG oscillations: from correlation to causality. Int. J. Psychophysiol. 103, 12–21. 10.1016/j.ijpsycho.2015.02.003 25659527

[B75] Hiltunen T. Kantola J. Elseoud A. A. Lepola P. Suominen K. Starck T.. (2014). Infra-slow EEG fluctuations are correlated with resting-state network dynamics in fMRI. J. Neurosci. 34, 356–362. 10.1523/JNEUROSCI.0276-13.2014 24403137 PMC6608153

[B76] Holz E. M. Glennon M. Prendergast K. Sauseng P. (2010). Theta-gamma phase synchronization during memory matching in visual working memory. Neuroimage 52, 326–335. 10.1016/j.neuroimage.2010.04.003 20382239

[B77] Hoy K. E. Bailey N. Arnold S. Windsor K. John J. Daskalakis Z. J.. (2015). The effect of γ-tACS on working memory performance in healthy controls. Brain Cogn. 101, 51–56. 10.1016/j.bandc.2015.11.002 26580743

[B78] Hoy K. E. Whitty D. Bailey N. Fitzgerald P. B. (2016). Preliminary investigation of the effects of γ -tACS on working memory in schizophrenia. J. Neural Transm. 123, 1205–1212. 10.1007/s00702-016-1554-1 27116682

[B79] Hsieh L. T. Ranganath C. (2014). Frontal midline theta oscillations during working memory maintenance and episodic encoding and retrieval. Neuroimage 85, 721–729. 10.1016/j.neuroimage.2013.08.003 23933041 PMC3859771

[B80] Hulme C. Melby-Lervåg M. (2012). Current evidence does not support the claims made for CogMed working memory training. J. Appl. Res. Mem. Cogn. 1, 197–200. 10.1016/j.jarmac.2012.06.006

[B81] Jaeggi S. M. Buschkuehl M. Jonides J. Perrig W. J. (2008). Improving fluid intelligence with training on working memory. Proc. Natl. Acad. Sci. U.S.A. 105, 6829–6833. 10.1073/pnas.0801268105 18443283 PMC2383929

[B82] Jaušovec N. Jaušovec K. (2014). Increasing working memory capacity with theta transcranial alternating current stimulation (tACS). Biol. Psychol. 96, 42–47. 10.1016/j.biopsycho.2013.11.006 24291565

[B83] Jaušovec N. Jaušovec K. Pahor A. (2014). The influence of theta transcranial alternating current stimulation (tACS) on working memory storage and processing functions. Acta Psychol. 146, 1–6. 10.1016/j.actpsy.2013.11.011 24361739

[B84] Jensen O. Colgin L. L. (2007). Cross-frequency coupling between neuronal oscillations. Trends Cogn. Sci. 11, 267–269. 10.1016/j.tics.2007.05.003 17548233

[B85] Jensen O. Lisman J. E. (1996). Novel lists of 7±2 known items can be reliably stored in an oscillatory short-term memory network: interaction with long-term memory. Learn. Mem. 3, 257–263. 10.1101/lm.3.2-3.257 10456095

[B86] Jensen O. Spaak E. Zumer J. M. (2014). Human brain oscillations: From physiological mechanisms to analysis and cognition, in Magnetoencephalography: From Signals to Dynamic Cortical Networks, eds Supek S. Aine C. J. (Berlin: Springer-Verlag), 359–403. 10.1007/978-3-642-33045-2_17

[B87] Jeong J. (2004). EEG dynamics in patients with Alzheimer's disease. Clin. Neurophysiol. 115, 1490–1505. 10.1016/j.clinph.2004.01.001 15203050

[B88] Jirsa V. Müller V. (2013). Cross-frequency coupling in real and virtual brain networks. Front. Comput. Neurosci. 7:78. 10.3389/fncom.2013.00078 23840188 PMC3699761

[B89] Jones K. T. Arciniega H. Berryhill M. E. (2019). Replacing tDCS with theta tACS provides selective, but not general WM benefits. Brain Res. 1720:146324. 10.1016/j.brainres.2019.146324 31279843

[B90] Jones K. T. Johnson E. L. Berryhill M. E. (2020). Frontoparietal theta-gamma interactions track working memory enhancement with training and tDCS. Neuroimage 211:116615. 10.1016/j.neuroimage.2020.116615 32044440 PMC7733399

[B91] Kamiński J. Brzezicka A. Wróbel A. (2011). Short-term memory capacity (7±2) predicted by theta to gamma cycle length ratio. Neurobiol. Learn. Mem. 95, 19–23. 10.1016/j.nlm.2010.10.001 20951219

[B92] Kane M. J. Brown L. H. McVay J. C. Silvia P. J. Myin-Germeys I. Kwapil T. R. (2007). For whom the mind wanders, and when: an experience-sampling study of working memory and executive control in daily life. Psychol. Sci. 18, 614–621. 10.1111/j.1467-9280.2007.01948.x 17614870

[B93] Kane M. J. Tuholski S. W. Hambrick D. Z. Wilhelm O. Payne T. W. Engle R. W. (2004). The generality of working memory capacity: a latent-variable approach to verbal and visuospatial memory span and reasoning. J. Exp. Psychol. Gen. 133, 189–217. 10.1037/0096-3445.133.2.189 15149250

[B94] Kehler L. Francisco C. O. Uehara M. A. Moussavi Z. (2020). The effect of transcranial alternating current stimulation (tACS) on cognitive function in older adults with dementia, in Proceedings of the Annual International Conference of the IEEE Engineering in Medicine and Biology Society, EMBS (Institute of Electrical and Electronics Engineers Inc.), 3649–3653. 10.1109/EMBC44109.2020.9175903 33018792

[B95] Klimesch W. (1999). EEG alpha and theta oscillations reflect cognitive and memory performance: a review and analysis. Brain Res. Rev. 29, 169–195. 10.1016/S0165-0173(98)00056-3 10209231

[B96] Klimesch W. (2018). The frequency architecture of brain and brain body oscillations: an analysis. Eur. J. Neurosci. 48, 2431–2453. 10.1111/ejn.14192 30281858 PMC6668003

[B97] Klingberg T. (2010). Training and plasticity of working memory. Trends Cogn. Sci. 14, 317–324. 10.1016/j.tics.2010.05.002 20630350

[B98] Klingberg T. Forssberg H. Westerberg H. (2002). Training of working memory in children with ADHD. J. Clin. Exp. Neuropsychol. 24, 781–791. 10.1076/jcen.24.6.781.8395 12424652

[B99] Knyazev G. G. Slobodskoj-Plusnin J. Y. Bocharov A. V. Pylkova L. V. (2011). The default mode network and EEG alpha oscillations: an independent component analysis. Brain Res. 1402, 67–79. 10.1016/j.brainres.2011.05.052 21683942

[B100] Köster M. Friese U. Schöne B. Trujillo-Barreto N. Gruber T. (2014). Theta-gamma coupling during episodic retrieval in the human EEG. Brain Res. 1577, 57–68. 10.1016/j.brainres.2014.06.028 24978601

[B101] Kuhnke P. Meyer L. Friederici A. D. Hartwigsen G. (2017). Left posterior inferior frontal gyrus is causally involved in reordering during sentence processing. Neuroimage 148, 254–263. 10.1016/j.neuroimage.2017.01.013 28069544

[B102] Lakatos P. Shah A. S. Knuth K. H. Ulbert I. Karmos G. Schroeder C. E. (2005). An oscillatory hierarchy controlling neuronal excitability and stimulus processing in the auditory cortex. J. Neurophysiol. 94, 1904–1911. 10.1152/jn.00263.2005 15901760

[B103] Lara G. A. Alekseichuk I. Turi Z. Lehr A. Antal A. Paulus W. (2018). Perturbation of theta-gamma coupling at the temporal lobe hinders verbal declarative memory. Brain Stimul. 11, 509–517. 10.1016/j.brs.2017.12.007 29317186

[B104] Lee Y. Y. Yang C. Y. (2014). Utilizing the extent of theta–gamma synchronization to estimate visuospatial memory ability. Australas. Phys. Eng. Sci. Med. 37, 665–672. 10.1007/s13246-014-0299-0 25217964

[B105] Leszczyński M. Fell J. Axmacher N. (2015). Rhythmic working memory activation in the human hippocampus. Cell Rep. 13, 1272–1282. 10.1016/j.celrep.2015.09.081 26527004

[B106] Lisman J. E. Idiart M. A. P. (1995). Storage of 7 ± 2 short-term memories in oscillatory subcycles. Science 267, 1512–1515. 10.1126/science.7878473 7878473

[B107] Lisman J. E. Jensen O. (2013). The theta-gamma neural code. Neuron 77, 1002–1016. 10.1016/j.neuron.2013.03.007 23522038 PMC3648857

[B108] López-Alonso V. Cheeran B. Río-Rodríguez D. Fernández-Del-Olmo M. (2014). Inter-individual variability in response to non-invasive brain stimulation paradigms. Brain Stimul. 7, 372–380. 10.1016/j.brs.2014.02.004 24630849

[B109] Luo W. Guan J.-S. (2018). Do brain oscillations orchestrate memory? Brain Sci. Adv. 4, 16–33. 10.26599/BSA.2018.9050008

[B110] Lynn P. A. Sponheim S. R. (2016). Disturbed theta and gamma coupling as a potential mechanism for visuospatial working memory dysfunction in people with schizophrenia. Neuropsychiatr. Electrophysiol. 2, 1–30. 10.1186/s40810-016-0022-3

[B111] MacOveanu J. Klingberg T. Tegnér J. (2007). Neuronal firing rates account for distractor effects on mnemonic accuracy in a visuo-spatial working memory task. Biol. Cybern. 96, 407–419. 10.1007/s00422-006-0139-8 17260154

[B112] Malenínská K. Rudolfová V. Šulcová K. Koudelka V. Brunovský M. Horáček J.. (2021). Is short-term memory capacity (7±2) really predicted by theta to gamma cycle length ratio? Behav. Brain Res. 414:113465. 10.1016/j.bbr.2021.113465 34265319

[B113] Mann E. O. Paulsen O. (2005). Mechanisms underlying gamma ('40 Hz') network oscillations in the hippocampus - a mini-review. Prog. Biophys. Mol. Biol. 87, 67–76. 10.1016/j.pbiomolbio.2004.06.004 15471591

[B114] Maris E. van Vugt M. Kahana M. (2011). Spatially distributed patterns of oscillatory coupling between high-frequency amplitudes and low-frequency phases in human iEEG. Neuroimage 54, 836–850. 10.1016/j.neuroimage.2010.09.029 20851192

[B115] Marzetti L. Basti A. Chella F. D'Andrea A. Syrjälä J. Pizzella V. (2019). Brain functional connectivity through phase coupling of neuronal oscillations: a perspective from magnetoencephalography. Front. Neurosci. 13:964. 10.3389/fnins.2019.00964 31572116 PMC6751382

[B116] Merker B. (2013). Cortical gamma oscillations: the functional key is activation, not cognition. Neurosci. Biobehav. Rev. 37, 401–417. 10.1016/j.neubiorev.2013.01.013 23333264

[B117] Miniussi C. Ruzzoli M. (2013). Transcranial stimulation and cognition. Handb. Clin. Neurol. 116, 739–750. 10.1016/B978-0-444-53497-2.00056-5 24112935

[B118] Mizuhara H. Yamaguchi Y. (2011). Neuronal ensemble for visual working memory via interplay of slow and fast oscillations. Eur. J. Neurosci. 33, 1925–1934. 10.1111/j.1460-9568.2011.07681.x 21488989

[B119] Mormann F. Fell J. Axmacher N. Weber B. Lehnertz K. Elger C. E.. (2005). Phase/amplitude reset and theta–gamma interaction in the human medial temporal lobe during a continuous word recognition memory task. Hippocampus 15, 890–900. 10.1002/hipo.20117 16114010

[B120] Nadel L. Hardt O. (2011). Update on memory systems and processes. Neuropsychopharmacology 36, 251–273. 10.1038/npp.2010.169 20861829 PMC3055510

[B121] Niebur E. (2002). Electrophysiological correlates of synchronous neural activity and attention: a short review. BioSystems 67, 157–166. 10.1016/S0303-2647(02)00102-8 12459295

[B122] Papazova I. Strube W. Hoffmann L. Schwippel T. Padberg F. Palm U.. (2020). T54 effects of gamma transcranial alternating current stimulation to the left dorsolateral prefrontal cortex on working memory in schizophrenia patients. Schizophr. Bull. 46, S251–S252. 10.1093/schbul/sbaa029.614

[B123] Park H. Lee D. S. Kang E. Kang H. Hahm J. Kim J. S.. (2016). Formation of visual memories controlled by gamma power phase-locked to alpha oscillations. Sci. Rep. 6:28092. 10.1038/srep28092 27306959 PMC4910116

[B124] Park J. Y. Jhung K. Lee J. An S. K. (2013). Theta-gamma coupling during a working memory task as compared to a simple vigilance task. Neurosci. Lett. 532, 39–43. 10.1016/j.neulet.2012.10.061 23149131

[B125] Park J. Y. Lee Y. R. Lee J. (2011). The relationship between theta-gamma coupling and spatial memory ability in older adults. Neurosci. Lett. 498, 37–41. 10.1016/j.neulet.2011.04.056 21571037

[B126] Persuh M. Larock E. Berger J. (2018). Working memory and consciousness: the current state of play. Front. Hum. Neurosci. 12:78. 10.3389/fnhum.2018.00078 29551967 PMC5840147

[B127] Pinal D. Zurrón M. Díaz F. Sauseng P. (2015). Stuck in default mode: inefficient cross-frequency synchronization may lead to age-related short-term memory decline. Neurobiol. Aging 36, 1611–1618. 10.1016/j.neurobiolaging.2015.01.009 25670334

[B128] Popov T. Jensen O. Schoffelen J. M. (2018). Dorsal and ventral cortices are coupled by cross-frequency interactions during working memory. Neuroimage 178, 277–286. 10.1016/j.neuroimage.2018.05.054 29803957

[B129] Rajji T. K. Zomorrodi R. Barr M. S. Blumberger D. M. Mulsant B. H. Daskalakis Z. J. (2017). Ordering information in working memory and modulation of gamma by theta oscillations in humans. Cereb. Cortex 27, 1482–1490. 10.1093/cercor/bhv326 26759480

[B130] Riddle J. McFerren A. Frohlich F. (2021). Causal role of cross-frequency coupling in distinct components of cognitive control. Prog. Neurobiol. 202:102033. 10.1016/j.pneurobio.2021.102033 33741402 PMC8184612

[B131] Rizzuto D. S. Madsen J. R. Bromfield E. B. Schulze-Bonhage A. Kahana M. J. (2006). Human neocortical oscillations exhibit theta phase differences between encoding and retrieval. Neuroimage 31, 1352–1358. 10.1016/j.neuroimage.2006.01.009 16542856

[B132] Roberts B. M. Hsieh L. T. Ranganath C. (2013). Oscillatory activity during maintenance of spatial and temporal information in working memory. Neuropsychologia 51, 349–357. 10.1016/j.neuropsychologia.2012.10.009 23084981 PMC3546228

[B133] Rodriguez-Larios J. Alaerts K. (2019). Tracking transient changes in the neural frequency architecture: harmonic relationships between theta and alpha peaks facilitate cognitive performance. J. Neurosci. 39, 6291–6298. 10.1523/JNEUROSCI.2919-18.2019 31175211 PMC6687903

[B134] Romei V. Driver J. Schyns P. G. Thut G. (2011). Rhythmic TMS over parietal cortex links distinct brain frequencies to global versus local visual processing. Curr. Biol. 21, 334–337. 10.1016/j.cub.2011.01.035 21315592 PMC3063337

[B135] Roux F. Uhlhaas P. J. (2014). Working memory and neural oscillations: Alpha-gamma versus theta-gamma codes for distinct WM information? Trends Cogn. Sci. 18, 16–25. 10.1016/j.tics.2013.10.010 24268290

[B136] Salimpour Y. Anderson W. S. (2019). Cross-Frequency coupling based neuromodulation for treating neurological disorders. Front. Neurosci. 13:125. 10.3389/fnins.2019.00125 30846925 PMC6393401

[B137] Sauseng P. Griesmayr B. Freunberger R. Klimesch W. (2010). Control mechanisms in working memory: a possible function of EEG theta oscillations. Neurosci. Biobehav. Rev. 34, 1015–1022. 10.1016/j.neubiorev.2009.12.006 20006645

[B138] Sauseng P. Klimesch W. Doppelmayr M. Pecherstorfer T. Freunberger R. Hanslmayr S. (2005). EEG alpha synchronization and functional coupling during top-down processing in a working memory task. Hum. Brain Mapp. 26, 148–155. 10.1002/hbm.20150 15929084 PMC6871735

[B139] Sauseng P. Klimesch W. Heise K. F. Gruber W. R. Holz E. Karim A. A.. (2009). Brain oscillatory substrates of visual short-term memory capacity. Curr. Biol. 19, 1846–1852. 10.1016/j.cub.2009.08.062 19913428

[B140] Sauseng P. Peylo C. Biel A. L. Friedrich E. V. C. Romberg-Taylor C. (2019). Does cross-frequency phase coupling of oscillatory brain activity contribute to a better understanding of visual working memory? Br. J. Psychol. 110, 245–255. 10.1111/bjop.12340 30079531

[B141] Schack B. Vath N. Petsche H. Geissler H. G. Möller E. (2002). Phase-coupling of theta-gamma EEG rhythms during short-term memory processing. Int. J. Psychophysiol. 44, 143–163. 10.1016/S0167-8760(01)00199-4 11909647

[B142] Sederberg P. B. Kahana M. J. Howard M. W. Donner E. J. Madsen J. R. (2003). Theta and gamma oscillations during encoding predict subsequent recall. J. Neurosci. 23, 10809–10814. 10.1523/JNEUROSCI.23-34-10809.2003 14645473 PMC6740970

[B143] Siebenhühner F. Wang S. H. Arnulfo G. Lampinen A. Nobili L. Palva J. M.. (2020). Genuine cross-frequency coupling networks in human resting-state electrophysiological recordings. PLoS Biol. 18:e3000685. 10.1371/journal.pbio.3000685 32374723 PMC7233600

[B144] Siebenhühner F. Wang S. H. Palva J. M. Palva S. (2016). Cross-frequency synchronization connects networks of fast and slow oscillations during visual working memory maintenance. Elife 5:e36. 10.7554/eLife.13451.036 27669146 PMC5070951

[B145] Siegel M. Donner T. H. Engel A. K. (2012). Spectral fingerprints of large-scale neuronal interactions. Nat. Rev. Neurosci. 13, 121–134. 10.1038/nrn3137 22233726

[B146] Siems M. Siegel M. (2020). Dissociated neuronal phase- and amplitude-coupling patterns in the human brain. Neuroimage 209:116538. 10.1016/j.neuroimage.2020.116538 31935522 PMC7068703

[B147] Smith E. H. Banks G. P. Mikell C. B. Cash S. S. Patel S. R. Eskandar E. N.. (2015). Frequency-Dependent representation of reinforcement-related information in the human medial and lateral prefrontal cortex. J. Neurosci. 35:15827. 10.1523/JNEUROSCI.1864-15.2015 26631465 PMC4666912

[B148] Sotero R. C. (2016). Topology, cross-frequency, and same-frequency band interactions shape the generation of phase-amplitude coupling in a neural mass model of a cortical column. PLoS Comput. Biol. 12:1005180. 10.1371/journal.pcbi.1005180 27802274 PMC5089773

[B149] Sreeraj V. S. Shanbhag V. Nawani H. Shivakumar V. Damodharan D. Bose A.. (2017). Feasibility of online neuromodulation using transcranial alternating current stimulation in schizophrenia. Indian J. Psychol. Med. 39:92. 10.4103/0253-7176.198937 28250567 PMC5330000

[B150] Tang W. Liu H. Douw L. Kramer M. A. Eden U. T. Hämäläinen M. S.. (2017). Dynamic connectivity modulates local activity in the core regions of the default-mode network. Proc. Natl. Acad. Sci. U.S.A. 114, 9713–9718. 10.1073/pnas.1702027114 28827337 PMC5594646

[B151] Thut G. Miniussi C. (2009). New insights into rhythmic brain activity from TMS-EEG studies. Trends Cogn. Sci. 13, 182–189. 10.1016/j.tics.2009.01.004 19286414

[B152] Tseng Y.-L. Liu H.-H. Liou M. Tsai A. C. Chien V. S. C. Shyu S.-T.. (2019). Lingering sound: event-related phase-amplitude coupling and phase-locking in fronto-temporo-parietal functional networks during memory retrieval of music melodies. Front. Hum. Neurosci. 13:150. 10.3389/fnhum.2019.00150 31178706 PMC6538802

[B153] Turi Z. Mittner M. Lehr A. Bürger H. Antal A. Paulus W. (2020). θ-γ cross-frequency transcranial alternating current stimulation over the trough impairs cognitive control. eNeuro 7, 1–12. 10.1523/ENEURO.0126-20.2020 32764077 PMC7540931

[B154] Unsworth N. Engle R. W. (2007). On the division of short-term and working memory: an examination of simple and complex span and their relation to higher order abilities. Psychol. Bull. 133, 1038–1066. 10.1037/0033-2909.133.6.1038 17967093

[B155] van der Meij R. Kahana M. Maris E. (2012). Phase-amplitude coupling in human electrocorticography is spatially distributed and phase diverse. J. Neurosci. 32, 111–123. 10.1523/JNEUROSCI.4816-11.2012 22219274 PMC6621324

[B156] Van Vugt M. K. Chakravarthi R. Lachaux J.-P. (2014). For whom the bell tolls: periodic reactivation of sensory cortex in the gamma band as a substrate of visual working memory maintenance. Front. Hum. Neurosci. 8:696. 10.3389/fnhum.2014.00696 25237304 PMC4154390

[B157] Veniero D. Vossen A. Gross J. Thut G. (2015). Lasting EEG/MEG aftereffects of rhythmic transcranial brain stimulation: level of control over oscillatory network activity. Front. Cell. Neurosci. 9:477. 10.3389/fncel.2015.00477 26696834 PMC4678227

[B158] Vilberg K. L. Rugg M. D. (2008). Memory retrieval and the parietal cortex: a review of evidence from a dual-process perspective. Neuropsychologia 46, 1787–1799. 10.1016/j.neuropsychologia.2008.01.004 18343462 PMC2488316

[B159] Vogel E. K. Woodman G. F. Luck S. J. (2001). Storage of features, conjunctions, and objects in visual working memory. J. Exp. Psychol. Hum. Percept. Perform. 27, 92–114. 10.1037/0096-1523.27.1.92 11248943

[B160] Vosskuhl J. Huster R. J. Herrmann C. S. (2015). Increase in short-term memory capacity induced by down-regulating individual theta frequency via transcranial alternating current stimulation. Front. Hum. Neurosci. 9:257. 10.3389/fnhum.2015.00257 26005411 PMC4424841

[B161] Voytek B. Canolty R. T. Shestyuk A. Crone N. E. Parvizi J. Knight R. T. (2010). Shifts in gamma phase-amplitude coupling frequency from theta to alpha over posterior cortex during visual tasks. Front. Hum. Neurosci. 4:191. 10.3389/fnhum.2010.00191 21060716 PMC2972699

[B162] Walsh V. Cowey A. (2000). Transcranial magnetic stimulation and cognitive neuroscience. Nat. Rev. Neurosci. 1, 73–80. 10.1038/35036239 11252771

[B163] Wang J. Fang Y. Wang X. Yang H. Yu X. Wang H. (2017). Enhanced gamma activity and cross-frequency interaction of resting-state electroencephalographic oscillations in patients with Alzheimer's disease. Front. Aging Neurosci. 9:243. 10.3389/fnagi.2017.00243 28798683 PMC5526997

[B164] Weiler M. Teixeira C. V. L. Nogueira M. H. De Campos B. M. Damasceno B. P. Cendes F.. (2014). Differences and the relationship in default mode network intrinsic activity and functional connectivity in mild alzheimer's disease and amnestic mild cognitive impairment. Brain Connect. 4, 567–574. 10.1089/brain.2014.0234 25026537 PMC4202997

[B165] Wolinski N. Cooper N. R. Sauseng P. Romei V. (2018). The speed of parietal theta frequency drives visuospatial working memory capacity. PLoS Biol. 16:e2005348. 10.1371/journal.pbio.2005348 29538384 PMC5868840

[B166] Zhang H. Y. Wang S. J. Xing J. Liu B. Ma Z. L. Yang M.. (2009). Detection of PCC functional connectivity characteristics in resting-state fMRI in mild Alzheimer's disease. Behav. Brain Res. 197, 103–108. 10.1016/j.bbr.2008.08.012 18786570

[B167] Ziemann U. Siebner H. R. (2015). Inter-subject and intersession variability of plasticity induction by non-invasive brain stimulation: boon or bane? Brain Stimul. 8, 662–663. 10.1016/j.brs.2015.01.409 25704341

